# Involving the parents of preterm babies

**Published:** 2017

**Authors:** Julie Flanagan, Erika Maka, Cristina Nitulescu

**Affiliations:** Retinopathy of Prematurity Screening Co-ordinator: Newborn Intensive Care Unit, St Marys Hospital, Central Manchester University Hospitals NHS Foundation Trust, Manchester, UK.; Ophthalmologist and Paediatric Ophthalmologist: Semmelweis University, Maria Str. 39, Budapest, Hungary.; Senior ophthalmologist: Oftapro Ophthalmology Clinic and Institute for Mother and Child Health, Bucharest, Romania.

**Figure:**
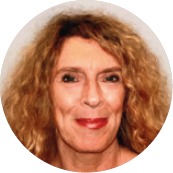
Julie Flanagan

**Figure:**
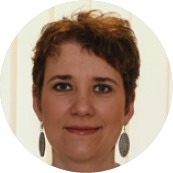
Erika Maka

**Figure:**
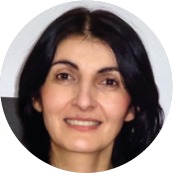
Cristina Nitulescu

**The prevention, detection and treatment of ROP is a team responsibility. Parents are important members of the team and their involvement is essential in ensuring optimal visual outcomes.**

**Figure:**
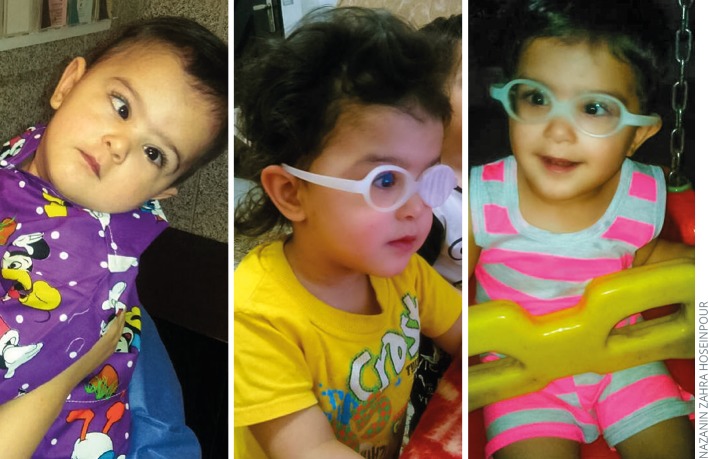
Thanks to excellent teamwork between parents and the medical team, a child with complications of ROP can now see well. IRAN

In the hospital, neonatologists, paediatricians, ophthalmologists, nurses and other allied health professionals are all involved in the care, screening and treatment of a baby with ROP. In such a busy clinical setting, it is easy to forget that parents are also important members of the team.

The role of parents must not be underestimated.^1^ Not only can they help to prevent ROP in the clinic, they are also responsible for bringing their child back for screening and treatment appointments. Without parents' active involvement, ROP can have devastating consequences.

Good communication is at the centre of developing positive and productive relationships with parents, and it must start from the day a preterm infant is admitted to the neonatal unit. The members of the medical team can involve parents by communicating clearly and simply about the care their baby needs and how the parents can help. This builds a relationship of trust. It also helps parents to feel part of the team and to understand the important role they can play.

**The art of good communication**

Parents of preterm infants are likely to be very anxious: they are in an unfamiliar environment and the health of their child can change rapidly. They may have other children or dependants at home and may not be able to spend much time in the neonatal unit. Parents often blame themselves for their child's condition and can feel helpless.

All our communication should be kind and understanding. Talk *with* parents and not *at* them. It is important to find out what they already know about prematurity, vision and the eye as this can provide the basis for communication. Simple, clear language is very important, so that what we say is understandable. Ask whether parents have any questions, and allow plenty of time for them to respond. Information may have to be repeated several times and may also change as the situation changes and the parents learn more, or are asked to do more.

Answering all questions without hesitation enhances parents' trust. We should let parents know that they can ask questions and can express their worries and concerns at any time. Empathy, listening and patience are essential in good communication, as is good eye contacta. Wait until a parent has finished before you reply; interrupting can prevent parents from asking important questions or sharing important information with the medical team. Parents like to know the simple truth.

There are also practical things parents can do to help. Supportive care practices such as kangaroo care and feeding babies breast milk (pp. 50–54) not only improve health outcomes in preterm babies, but also involves the parents in a very positive way.

## The parents' perspective

When the medical team's focus is on ensuring the survival of a preterm baby, it may be quite difficult to see the situation from a parent's perspective. Admission to a neonatal intensive care unit makes parenting very difficult: tubes, monitors and incubators can get in the way of the normal bonding between parent and child. Family-centred or family-integrated care (**http://familyintegratedcare.com**) is an approach that has been developed to enable parents to have the close interaction and contact they need with their infant^1^; this also includes kangaroo care and feeding babies breast milk (from the breast or using a cup).

Supportive care practices and approaches can change the relationship between the medical team and parents by emphasising – to both parents and the team – the importance of working together. Many studies report significant improvement in clinical outcomes as a consequence of adopting these practices.^2^,^3^ Studies have shown that parents who are more aware of their child's medical condition, and who are engaged in their care while in the neonatal intensive care unit, have more positive attitudes and are more likely to bring their infant back for follow-up.^4^,^5^

Educating parents and increasing their awareness about ROP should start weeks before the first screening appointment is planned. Parents need to be given time to absorb the information and to ask questions or express their concerns, and do so at their own pace. Nurses are ideally placed to start talking to parents about the potential complications of prematurity, including ROP and other visual complications (pp. 62–64). It is important to take into account the amount of information each parent wants at any specific time. Appointing an experienced nurse as an ROP co-ordinator can further enhance nurse-parent communication.^6^ When it is time for ROP screening, a nurse or neonatologist who knows the parents well can introduce the ophthalmologist, as this builds trust.

For many infants, ROP screening starts in the neonatal unit but continues after they have been sent home with their parents. Parents need to understand the importance and the timing of screening so that they will bring their child back at the right time. They also need to know that laser treatment, if needed, cannot be delayed. Infants who have had treatment need regular follow-up visits to ensure that the treatment has been successful in the short term, and to detect and manage complications in the longer term (pp. 62–64). The active engagement of parents can, therefore, make all the difference between success and failure in preserving their child's vision.

## Supporting communication

Written and visual materials can help to support verbal communication. Posters which use simple language and clear images can be used to explain the importance of ROP screening, and that treatment may be needed. (Note: The images in this issue can be used for posters and other educational materials, except if there is a copyright notice. Visit **www.flickr.com/communityeyehealth** to download high-resolution images.)

If parents are given a booklet when their baby is admitted, ensure that ROP is mentioned. This provides a gradual introduction to ROP in the first few days after admission.

Each unit should have a parent information booklet about ROP which parents can read themselves and which staff can use as the basis of education and counselling. The booklet should use simple terminology and provide consistent information which the team can refer back to if required. Highlight the fact that the risk of ROP can be reduced and that treatment (if needed) is usually successful.

Cover the following topics:

- What is ROP and why does it occur?
- How common is ROP?
- How is ROP detected?
- What should we expect during and after an eye examination?
- Will my child need treatment?
- What happens if my baby is unwell?
- Where can I find out more? (with websites if appropriate)
- Contact details for members of the neonatal team or the ophthalmologist

**Figure 1:**
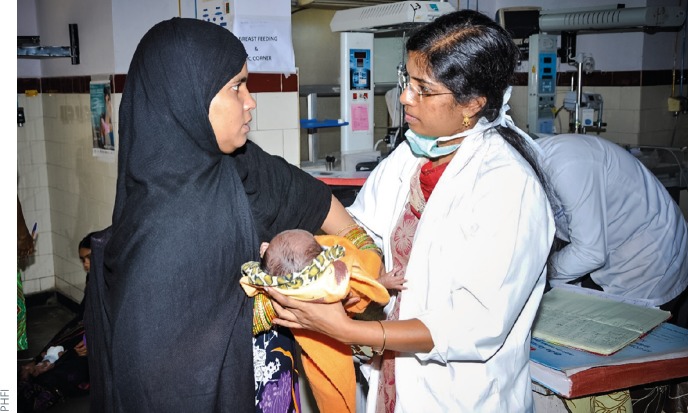
An ophthalmologist explains to the mother the importance of screening a preterm baby.

Images of the normal retina and the retina with ROP can be a very useful way to educate parents about ROP.

## Systems to support parental involvement

The ophthalmologist is the best person to communicate the findings of screening to parents, accompanied by a member of neonatal team whom the parents already know (usually a nurse). The ophthalmologist should explain what the findings mean using clear, non-technical language (Figure 1). The nurse can provide additional information if the parents have questions or concerns after the ophthalmologist has left the unit.

Maintaining good medical records of the findings of screening, the management decision, and any follow-up (see p. 58), is essential. Note whether parents were informed personally about the findings and what happens next. Good medical records also enhance team communication. The consultant neonatologist or paediatrician is responsible for co-ordinating follow-up screening, either in the neonatal unit or the eye department after discharge. This responsibility can be delegated to the ROP nurse co-ordinator, if one is in post, or a nurse.

If a baby is to be discharged from the unit before ROP screening has been completed, it is crucial that the first follow-up appointment is made before the family leaves. The neonatal team must have the correct contact details for the family, i.e., their address and two up-to-date mobile numbers, so that they can be reminded about about the next appointment and contacted immediately if they do not attend for screening or treatment.

Give parents the following information:

- The appointment date, time and place (which may be in the unit or in the hospital where the ophthalmologist works)
- Who to contact if there are problems
- Details about transport assistance or reimbursement of costs for travel, if appropriate
- Information about the consequences of late screening and the potential risk of blindness if screening does not take place.

This must be written in the child's medical records and in the discharge summary (which the parents keep).

In conclusion, good communication is an art which can be improved. It supports parental involvement, which in turn contributes to good medical care and better outcomes for a baby with ROP.
